# An analysis of the etiologies and economic indexes of inpatients with stage 5 chronic kidney disease in North China

**DOI:** 10.3389/fpubh.2022.956463

**Published:** 2022-12-01

**Authors:** Shan-Shan Guo, Yan-Li Gou, Jian-Dong Li, Hong-Fang Zhang, Yang Huang, Xi-Jie Zheng, Hang Chen, Zhun Sui

**Affiliations:** ^1^Department of Nephrology, Affiliated Hospital of Hebei University, Baoding, China; ^2^Tianjin Beichen Center for Disease Control and Prevention, Tianjin, China; ^3^Hebei University, Baoding, China; ^4^Department of Nephrology, Peking University People‘s Hospital, Beijing, China

**Keywords:** stage 5 CKD, hemodialysis, peritoneal dialysis, etiology analysis, economic burden

## Abstract

**Objective:**

The aim of this study was to analyze the epidemiological characteristics of the causes of chronic kidney disease (CKD) stage 5 patients in North China and to investigate the economic burden of those on hemodialysis (HD) or peritoneal dialysis (PD), as well as the associated influencing factors.

**Methods:**

General clinical information, etiological categories, and hospitalization costs for HD or PD were collected from 1,515 patients hospitalized with stage 5 CKD at the Affiliated Hospital of Hebei University from 2016 to 2018. Logistic regression analysis was used to analyze the independent influencing factors affecting patients' financial burden.

**Results:**

The highest rate of DN was found in patients aged 70 years or older (27.0%) and the highest incidence of primary glomerulopathy was found in patients aged <50 years (24.3%). Age, type of dialysis, and type of health insurance were independent influences on the total financial burden of patients, and the results of multifactorial logistic regression analysis showed that age [OR (95% CI): 1.009 (1.002, 1.020)] and type of dialysis [OR (95% CI): 1.746 (1.149, 2.659)] would increase the total financial burden. The type of health insurance would reduce the total financial burden [OR (95% CI): 0.222 (0.108, 0.418)].

**Conclusion:**

Chronic kidney disease, with its complex etiology and the heavy financial burden required for treatment, remains a more serious public health problem globally, and it is therefore necessary to further improve medical coverage for dialysis patients, increase management efforts, broaden pro-poor policies and increase the accessibility of medical services in low- and middle-income areas.

## Background

Chronic kidney disease (CKD) is defined as structural and functional disturbances of the kidney caused by various etiologies with a history of kidney damage of ≥3 months. The fifth stage of CKD is the end stage of irreversible decline in kidney function and is also known as end-stage renal disease (ESRD). In recent years, the prevention and treatment of CKD has become an important public health issue worldwide due to its increasing incidence and prevalence (1, 2). According to an epidemiological survey published in The Lancet, the prevalence of CKD in China exceeds 10.8% and the number of patients is estimated to reach 119.5 million. In recent years, in addition to the two most common causes of CKD, hypertension and diabetes, more causes have been identified that may contribute to CKD (3, 4), and because of the multiple causes of CKD and the interaction of various chronic diseases, it is difficult to contain or slow down the development of CKD from its causes. In addition to this, kidney damage is irreversible and kidney function gradually declines, making dialysis the main clinical treatment for ESRD (5–7). However, ESRD patients need to receive lifelong treatment, which imposes a heavy financial burden on the patients' families and society (8). In this study, information on 1,515 patients with stage 5 CKD admitted to the Hebei University Hospital from 2016 to 2018 was collected to analyze the economic burden of different treatments in order to reduce the economic burden on patients and the public health system, and to describe the epidemiological distribution of the associated etiology.

## Materials and methods

### Study subjects

Patients with five stages of CKD who were hospitalized at the Affiliated Hospital of Hebei University from January 1, 2016 to December 31, 2018 were selected to analyze the epidemiological characteristics and economic burden of their etiology. A total of 1,607 patients with stage 5 CKD were hospitalized during the period. One thousand one hundred and fifteen patients were included and 92 were excluded according to the inclusion and exclusion criteria. This study was approved by the Ethics Committee of the Affiliated Hospital of Hebei University to be conducted (HDFY-LL-2020-028).

Inclusion criteria were as follows: (i) no history of psychiatric disorders; (ii) complete clinical data; (iii) patients aged 18 years or older.

Exclusion criteria were as follows: (i) suffering from severe gastrointestinal diseases, serious infections, malignancies or other serious diseases; (ii) bedbound dialysis patients.

### Methods

The information of all 1,515 inpatients with stage 5 CKD was collected, and information cards were created for data collection. The collected case information included the demographic characteristics (gender, age, ethnicity), place of residence (urban/rural), type of surgical operation (indwelling catheter PD/HD access), hospitalization costs, type of health insurance, etc. The etiology of the patients was examined and classified in the clinical examination data of the subjects, and the data was collected and collated from the patients' clinical data, and the patients' etiology was clearly classified and pathologically supported in their clinical data. The etiological factors and economic burden of patients with stage 5 CKD were analyzed. In all hospitals from where data were extracted, they used digital data record systems, and they were all under the university using same variables in data record files.

### Statistical methods

SAS 9.4 statistical software was used for data analysis. The data were tested for normality before the statistical methods used to select the data for analysis. Two-person and two-computer independent data entry was performed using EpiData 3.1. Statistical descriptions of the count information were made using rates or composition ratios (%). For continuous data variables with skewed distribution, the median was used for statistical description. Comparisons of count data between multiple groups were made using the rank sum test and *P* < 0.05 was considered statistically significant. Independent influences on financial burden were analyzed using logistic regression analysis.

## Results

### The general characteristics

Among the 1,515 patients with stage 5 CKD, 55.7% were male, and 44.3% were female. The average age was 55.7 ± 13.6 years old, 99.3% were Han Chinese, 0.3% were Hui and 0.4% were Man. 51.2% from rural areas and 27.1% from urban areas. Five hundred and twenty seven patients (34.8%) underwent dialysis-related procedures, of whom 144 (9.5%) had indwelling catheter PD and 383 (25.3%) underwent HD access-related procedures. The details are shown in Table 1.

**Table 1:** The general characteristics of patients with stage 5 CKD (*n*, %).

	**Cases (*n*)**	**Composition ratio (%)**
**Gender**		
Male	844	55.7
Female	671	44.3
**Age (Years old)**		
<50	437	28.8
50–70	843	55.7
>70	235	15.5
**Ethnic**		
Han	1,505	99.3
Hui	4	0.3
Man	6	0.4
**Types of dialysis surgery procedures**		
Catheter indwelling for peritoneal dialysis	144	9.5
Hemodialysis access	383	25.3
No surgical operation	989	65.2
**The types of medical insurance**		
Medical insurance for urban employee	411	28.4
Medical insurance for urban residents	776	57.7
Poverty relief	1	0.1
Commercial medical insurance	179	11.8
Complete state medicine	6	0.4
Total pay at one's own expense	24	1.6

The hemodialysis access procedures included artificial arteriovenous fistula, central venous catheter indwelling, and semi-permanent catheter indwelling.

### Characteristics of the epidemiological distribution of the etiology of patients with CKD stage 5

Patients were divided into three groups according to age and the epidemiological distribution of causes between the groups was analyzed, as shown in Table 2.

**Table 2:** The etiological analysis of stage 5 CKD.

**The etiology of stage 5 CKD**	**Total**		<**50 years old**		**50–70 years old**		>**70 years old**		***p***
	**Cases**	**Percentage (%)**	**Cases**	**Percentage (%)**	**Cases**	**Percentage (%)**	**Cases**	**Percentage (%)**	
CKD of unknown etiology	388	25.6	128	29.8	216	26.1	45	19.7	<0.001
Diabetic retinopathy	320	21.1	80	18.7	186	22.5	62	27.3	<0.001
Hypertension-related renal injury	285	18.8	70	16.2	152	18.4	51	22.7	<0.001
Primary glomerulonephropathy	386	25.5	104	24.3	173	21.0	42	18.8	<0.001
Obstructive nephropathy	70	4.6	16	3.8	41	5.0	12	5.3	<0.001
Tumor-related nephropathy	53	3.5	12	2.6	27	3.2	9	3.8	<0.001
Others	13	0.9	20	4.6	31	3.8	5	2.4	–

Diabetic nephropathy: impaired kidney function caused by diabetes; hypertension-related kidney damage: impaired kidney function caused by hypertension; primary glomerulonephropathy: the most common glomerular disease, which can be clinically manifested as proteinuria, haematuria, oedema, and hypertension; obstructive nephropathy: refers to various causes of urinary tract obstruction, resulting in the inability of urine to drain normally and increased pressure above the obstruction, resulting in kidney dysfunction and substantial damage; tumor-related nephropathy: kidney damage caused by direct tumor invasion of the kidney, kidney damage caused by immune mechanism tissue and kidney damage caused by tumor metabolic abnormalities such as hyperuricaemia and hypercalcaemia.

The predominant age group of patients with stage 5 CKD of different etiologies was 50–70 years, followed by patients younger than 50 years, with the smallest proportion of patients older than 70 years.

Among the different age groups, excluding CKD of unknown etiology, the prevalence of diabetic nephropathy (DN), hypertension-related kidney injury, obstructive nephropathy and tumor-related nephropathy was significantly higher in patients aged >70 years than in other age groups, with prevalence rates of 27.3, 22.7, 5.3, and 3.8%, respectively. In this study, the rates of DN were found to be 18.0 and 23.0% in patients aged <50 years and 50–70 years, respectively, and the highest rate (27.0%) was found in patients aged 70 years or older. The incidence of primary glomerulopathy was significantly higher in patients aged <50 years than in other groups, with an incidence of 24.3%.

### Analysis of the economic burden of patients with stage 5 CKD

The median total medical cost of a single hospital stay for patients with stage 5 CKD was RMB 16,358.1, of which the median cost of Western medicine was RMB 6,064.5. There were 765 patients with no co-infections. Seven hundred and fifty patients (49.5%) developed infections and used antibiotics, with a median cost of RMB 1624.9 for antibiotic use.

#### Comparison of the financial burden on dialysis patients

Of the patients with stage 5 CKD, 526 underwent dialysis, 144 underwent indwelling catheter PD and 383 underwent HD access surgery. In this study, the medical costs of patients who underwent dialysis procedures were analyzed and the differences between the two groups were statistically significant (*p* < 0.05) in terms of the cost of Western medicine, disposable consumables and type of surgical operation. There was no statistically significant difference between the two groups in the costs of Chinese medicine, clinical diagnostics and laboratory tests as shown in Table 3.

**Table 3:** The medical expenditure in patients with dialysis.

**Cost**	**Types of dialysis surgery procedures**		***p***
	**Peritoneal dialysis M (Q1, Q3)**	**Hemodialysis M (Q1, Q3)**	
The total cost	24,924.88 (18796.07,32153.47)	20,843.88 (14438.98,30370.96)	0.0004
The cost of Western medicine	9,611.46 (7301.99,13045.93)	6,391.08 (4179.64,10132.28)	<0.0001
The cost of antibiotics	1,828.73 (1190.30,2749.65)	1,705.56 (906.30,2666.34)	0.2310
The cost of traditional Chinese medicine	446.23 (215.76,807.52)	345.77 (144.82,706.36)	0.2276
The cost of clinical diagnosis	930.00 (300.00,2145.00)	1,117.50 (577.50,1868.00)	0.5849
The cost of laboratory test	3,108.75 (2064.00,4122.00)	3,274.25 (2426.00,4357.50)	0.3989
The cost of image diagnosis	738.00 (538.00,1226.00)	930.00 (678.00,1354.00)	0.0010
The cost of disposable consumables	3,508.16 (3062.40,4254.75)	1,895.88 (888.42,4385.48)	<0.0001
The cost of surgery	132.30 (132.30,250.95)	135.66 (135.66,154.04)	0.0003

#### Univariate analysis of total economic burden on subjects

A univariate analysis of the total financial burden for all subjects showed that age, type of dialysis procedure and type of health insurance were independent influences on the total financial burden as shown in Table 4.

**Table 4:** Univariate analysis of total economic burden.

**Factor**	**Number of cases**	**Total economic burden**	**F**	***p***
Age			4.67	0.010
<50	437	18348.74 (15016.86, 22316.15)		
50–70	843	23779.68 (17531.26, 24835.68)		
>70	235	15043.71 (12516.57, 16538.43)		
Gender			0.793	0.230
Male	844	18346.93 (15634.98, 20122.63)		
Female	671	16112.15 (13346.51, 19264.75)		
Ethnicity			–	–
Han	1,505	16531.43 (12846.37, 20159.98)		
Hui	4	–		
Man	6	–		
Types of dialysis surgery			9.837	0.001
Peritoneal dialysis	144	24924.88 (18976.07, 32153.47)		
Hemodialysis	383	20843.88 (14438.98, 30370.96)		
No dialysis	989	14167.79 (12306.56, 18283.84)		
Type of medical insurance			4.988	0.001
Cities and towns	411	20478.53 (15126.31, 26128.26)		
Urban and rural	776	17531.62 (14876.15, 18876.34)		
Impoverished rescue	1	15637.23		
Commercial	179	15924.61 (14813.65, 16446.26)		
State-financed	6	24785.36 (18864.73, 26915.87)		
Self-paying	24	14753.16 (12685.87, 20186.74)		

#### Multi-factor analysis of the total economic burden on subjects

Based on the results of the univariate analysis, this study included age, type of dialysis procedure and type of health insurance in a multiple logistic regression model for analysis, which showed that age [OR (95% CI): 1.009 (1.002, 1.020)] and type of dialysis [OR (95% CI): 1.746 (1.149, 2.659)] had a There was a negative impact of Type of health insurance had a beneficial effect on total financial burden [OR (95% CI): 0.222 (0.108, 0.418)] as shown in Table 5.

**Table 5:** Multivariate analysis of total economic burden.

**Factor**	**Regression coefficient (β)**	**S.E**.	***P***	**OR (95%CI)**
Age	−0.633	0.174	0.0004	1.009 (1.002, 1.020)
Types of dialysis surgery	2.949	0.680	0.0093	1.746 (1.149, 2.659)
Type of medical insurance	3.301	0.662	<0.001	0.222 (0.108, 0.418)

## Discussion

### Characteristics of the etiological distribution of CKD patients

Domestic and international studies have shown that the epidemiological characteristics of CKD stage 5 vary from time to time and region to region (9). Influenced by environmental factors, lifestyle, economic conditions and level of treatment, the prevalence rate varies among regions and ethnic groups (10). In this study, patients with CKD in a specific time period were selected from the Affiliated Hospital of Hebei University. It was found that after excluding stage 5 CKD of unknown etiology among the subjects, the first and second most common etiologies among patients aged 70 years or older were DN and hypertension-related kidney injury, respectively, and primary glomerulonephritis was less common than the above two. As age decreases, DN and hypertension-related kidney injury gradually decrease, while primary glomerulonephritis gradually increases and ranks first. In patients under 50 years of age, primary glomerular disease remained the most common cause (25.5%), in line with national and international studies (11, 12). This study also showed that the proportion of hypertension-associated kidney injury increased progressively with age. The proportion of hypertension-related kidney injury ranked first among patients over 70 years of age (23%). Possible reasons for the increased incidence of tubular injury are (13, 14): more underlying disease, a natural decline in renal function with age, and increased use of contrast agents, antibiotics, NSAIDs and chemotherapy drugs in older patients (15, 16). In addition, as the incidence of tumors increases with age, tumor infiltration, metastasis, metabolic abnormalities, chemical administration and contrast agents may lead to impaired renal function, while conditions such as prostatic hypertrophy, urological and genital tumors or ureteral compression by metastatic pelvic tumors may lead to obstructive nephropathy (17), so greater attention should be paid to tumor-related nephropathy as well as obstructive nephropathy.

### Financial burden analysis

Dialysis imposes a huge financial burden on patients, and in recent years, with the increasing number of patients with CKD, the increasing number of patients developing CKD stage 5, and the application of new technologies and drugs, the upgrading of treatments for quality of life, the overall healthcare costs are increasing. The results of this study showed that mainly the cost of western drugs, the cost of disposable consumables and the cost of laboratory tests accounted for a greater proportion of the costs required for PD than HD. This is somewhat different from previous cost-effectiveness-based studies that concluded that PD was more cost-effective than HD, which may be due to the following reasons: (1) this study investigated the cost of a single hospital stay for stage 5 CKD, rather than the annual cost of maintenance dialysis (2) most of the patients undergoing the procedure were first-time RRT patients and disposable consumables, such as abdominal dialysis tubing, were more expensive; (3) the influence of the general national health policy environment, with lower drug costs and upward adjustments in the cost of medical consumables. As patients with CKD stage 5 require continuous dialysis, it is possible that PD is more cost effective than HD for the costs incurred by continuous dialysis, and we will continue to collect more data for further research. Based on the data collected so far, we further analyzed the relevant influences on the financial burden of patients with stage 5 CKD and found that age, dialysis and type of health insurance were independent influences on the financial burden of patients with stage 5 CKD. The effect of age on financial burden was small, with an OR value nearly equal to 1, indicating that age had almost no effect on financial burden. In contrast, dialysis and type of health insurance had a greater impact on financial burden, with dialysis patients experiencing a 0.746-fold increase in financial burden, while those using health insurance had a 0.222-fold increase in financial burden compared to those not using health insurance. Although various health insurance systems can be effective in reducing the financial burden of HD patients, patients still have to bear a greater financial hardship. For patients with stage 5 CKD, data from previous studies show that patients fail to choose the treatment that is most beneficial to their quality of survival, but rather the one with the least financial burden, given the direct financial burden (18). In low- and middle-income countries, dialysis reimbursement is not sufficient to treat all CKD patients, so there is a need to further improve health care coverage for dialysis patients, increase management efforts, broaden pro-poor policies and increase access to health care in low- and middle-income areas. However, we still have some limitations, such as no follow up visits, which will be conducted in future studies.

## Conclusion

Chronic kidney disease (CKD) affects more than 10% of the global population and poses a huge challenge to societies and healthcare systems worldwide (19). Different countries and localities can improve the affordability and accessibility of RRT (blood purification) for patients with stage 5 CKD by recommending scientifically effective, appropriate and affordable treatments based on their own circumstances (20). This study investigated the etiology and financial burden of patients with stage 5 CKD and found that the main financial burden of patients with stage 5 CKD in northern China comes from dialysis, while medical insurance and reimbursement can reduce the medical burden. However, at present, CKD patients in North China still bear a huge financial burden, and the state still does not take up enough of the financial share. The national government should push forward the reform of universal medical insurance in depth, establish a supplementary medical insurance system such as major medical insurance, implement medical assistance for serious illnesses, etc., and develop various forms of commercial health insurance to build up a multi-level and wide-ranging medical security system. It is hoped that this study will provide reference and ideas for the country to adjust the reimbursement system of medical insurance.

## Data availability statement

The raw data supporting the conclusions of this article will be made available by the authors, without undue reservation.

## Ethics statement

This study was conducted with approval from the Ethics Committee of the Affiliated Hospital of Hebei University (HDFY-LL-2020-028). The patients/participants provided their written informed consent to participate in this study.

## Author contributions

S-SG and Y-LG: conception and design of the research and writing of the manuscript. YH and J-DL: acquisition of data. X-JZ: analysis and interpretation of the data. H-FZ: statistical analysis. HC: obtaining financing and critical revision of the manuscript for intellectual content. All authors read and approved the final draft.

## Funding

This study was funded by the Key Research and Development Program of Hebei Province (No. 20377705D).

## Conflict of interest

The authors declare that the research was conducted in the absence of any commercial or financial relationships that could be construed as a potential conflict of interest.

## Publisher's note

All claims expressed in this article are solely those of the authors and do not necessarily represent those of their affiliated organizations, or those of the publisher, the editors and the reviewers. Any product that may be evaluated in this article, or claim that may be made by its manufacturer, is not guaranteed or endorsed by the publisher.
